# Impact of variable electricity price on heat pump operated buildings

**DOI:** 10.12688/openreseurope.15268.1

**Published:** 2022-12-07

**Authors:** Philipp Mascherbauer, Franziska Schöniger, Lukas Kranzl, Songmin Yu

**Affiliations:** 1 Energy Economics Group, Technische Universität Wien, Vienna, Vienna, 1040, Austria; 2 Fraunhofer Institute for Systems and Innovation Research, Karlsruhe, 76139, Germany

**Keywords:** demand-side management, heat pump, load shifting, optimization, building stock

## Abstract

**Background: **Residential buildings with heat pumps show promising possibilities for demand-side management. The operation optimization of such heating systems can lead to cost reduction and, at the same time, change electricity consumption patterns, which is especially prevalent in the case of a variable price signal. In this work, we deal with the following question: How does the volatility of a variable retail electricity price change the energy consumption of buildings with a smart energy management system?

**Methods: **In this context, we take Austria as an example and aggregate the findings of individual households to the national stock of single-family houses. This is done by simulating and optimzing heating operation in single representative buildings. The aggregation is done based on national building information statistics.

**Results: **This part of the Austrian building stock could shift 19.7 GWh of electricity per year through thermal inertia using a real-time electricity price from 2021. We show the future potential under the assumption of three electricity price trends for 2030, representing different decarbonisation ambition levels. The trends show that higher decarbonisation levels which lead to higher electricity prices increase the incentive to shift electric loads.

**Conclusions: **Real time pricing turns out to be  an effective incentive for buildings to shift electric loads by pre-heating the building mass. However, cost savings for individuals are relatively low which is why additional monetary incentives are needed to tap into that potential. Increased daily peak-to-peak demand from these buildings has to put into perspective to the overall grid load.

## Introduction

With increasing renewable power generation integrated into the power system, the fluctuations from the supply-side need to be balanced. Demand response (DR) can bring a valuable contribution to the power system by using time of use dependent pricing schemes ^1^. Residential buildings provide promising options for DR, especially the buildings with heat pumps (HP) and thermal storage that can be incentivized to shift loads by a price signal ^2^.

The operation of the HP can be optimized either by an in-house smart energy management system (SEMS) or a remote aggregator meaning that the heating system will be operated not by the homeowner. We will focus on an in-house operation implying that homeowners provide flexibility for their own benefit. In this paper we investigate one of many different incentives that can be used to influence the local control. One straightforward way is to introduce a variable real-time electricity price (RTP). If the prices in the following hours are known, the HP operation can be adapted to the price settings and economically optimize HP operation. Naturally, lower prices will occur when the production of renewable electricity is high, and prices will be higher when there are fewer renewables in the electricity mix ^3^.

Existing studies have evaluated the economic impacts of different pricing schemes on the HP-operated buildings ^4– 6^. In most studies, the flexibility is provided by the battery or thermal storage in the building. However, as shown in another strand of literature, the load shifting potential of the building’s thermal mass is also significant ^7, 8^.

In this study, we combine these two strands of literature and investigate how much the electricity consumption profile can be influenced if we combine variable electricity prices with the SEMS. We consider the flexibility of building mass alone and compare it to the flexibility with implemented thermal storage systems. Two research questions are formulated: How can RTP signals influence cost-minimizing control strategies of residential single family houses (SFH)? Second, what are the implications of variable real-time electricity pricing and in-house SEMS at the national level?

To answer these two questions, our study includes three parts:

- First, we developed an hourly technology operation model for individual households by taking a single SFH with a HP installed as an example. Assuming the electricity price, outside temperature, and radiation as input, the model determines the operation of the HP. The model can run in two modes. In the benchmark mode, the operation of technologies is simulated based on specific assumptions, with the energy consumption and cost calculated accordingly. In the optimization mode, technologies’ operation is optimized to minimize the energy cost.
- Second, we developed multiple scenarios for variable electricity prices following the RTP mechanism based on the Balmorel model ^9, 10^. The modeled prices reflect different scenarios with increasing marginal costs for power plants that provide flexibility. This way, we investigate how price volatility influences the incentive for load shifting.
- Third, by linking to the INVERT/EE-LAB ^11^ building stock model, we aggregate the results of individual households to the building stock level and explore the impact of RTP and in-house SEMS on the load shifting potential and the peak demand of the Austrian SFH building stock.

This paper contributes to existing literature by investigating load shifting potential on an hourly level over the whole year taking also future electricity price scenarios into account. Specifically the impact of increased price volatility is investigated. Additionally we show how load shifting measurements could influence the total peak demand on an aggregated level. The remainder of this paper is structured as follows. Section b overviews existing studies on HP-operated residential buildings’ flexibility potential and different variable electricity price mechanisms. Section b introduces our model in detail and provides the input data and scenario definitions. Then, in Section b, we present the results by analyzing the impact of various electricity prices on the load shifting behavior and the fiscal incentive. At last, we conclude in Section b, discuss the limitations of this approach, and point out the need for further research.

## Literature review

In literature, HP-operated buildings’ economic advantages with a variable price scheme are often discussed in combination with storage applications. Bechtel *et al.* ^12^ analyze how different buffer heat storage sizes and the HP power impact the cost savings of a HP-operated building with a variable price signal. They found that HP start-ups can be reduced significantly with higher storage capacity. Operation costs can be reduced by up to 20% with a 1500 l buffer storage in an SFH. However, when considering the additional investment costs for the larger storage, a 200 l buffer storage is preferred. The electricity prices used in this particular study were historical prices for the German-Luxemburg market (EPEX Spot). They do not consider different price schemes in this study. In a case study, Fitzpatrick *et al.* ^4^ compare three different electricity tariffs for a residential building with a combined HP and gas boiler. The house had a 2000 l buffer storage and a 400 l domestic hot water tank. It turned out that the building provided the highest flexibility with the RTP scheme. The other pricing schemes were a two-level day/night tariff and a critical peak pricing with three different price levels depending on the hour of the day. Patteeuw *et al.* ^6^ on the other hand, showed that dynamic time of use pricing performs inferior in comparison to the direct load control in regards to CO2 savings and cost savings with a high level of residential HP penetration. They assessed the value of load shifting in high-energy-efficient HP buildings. Two load shifting controls were adapted: 1) a direct load control and 2) dynamic time of use pricing. They analyzed how load shifting with residential HPs can reduce CO2 emissions and what incentive is best for home owners to participate in load shifting.

Ostergaard and Andersen ^5^ investigated the impact of electricity taxes on the incentive to increase HP power and thermal energy storage. Additionally, they examined the incentive of the alignment of HP operation with the dynamic electricity system needs in a district heating system. Variable taxes (hourly) incentivizes increasing thermal energy storage by 20%. The HP capacity however, was not affected. They did not consider different electricity pricing schemes.

Other studies denote that load shifting through variable electricity tariffs will lead to a higher electricity demand overall ^13, 14^. Miara *et al.* ^13^ stated that HP systems in combination with buffer storage provide promising DR possibilities. However, because of heat losses and the increased HP operating temperatures when charging the buffer storage, the electricity consumption increased by 20% when DR was fully exploited. Kelly *et al.* ^14^ tried to shift all heating loads from peak periods to off-peak periods in a typical detached dwelling in the UK. A 1000 l hot water storage was sufficient, but the overall electricity demand increased by 60%. Additionally, the tank’s charging demand was 50% higher than the average house would have had in the peak period.

Many studies investigating the impact of variable electricity prices focus primarily on load shifting with thermal or electrical storage. However, as widely discussed in existing studies, the thermal inertia of buildings also provides significant potential for load shifting.

For example, Golmohamadi ^15^ created a stochastic model predictive controller for HP buildings focusing on thermal inertia and heat storage in the building. They found that ambient temperature and electricity price uncertainty has a more significant impact on the economic analysis than the domestic hot water consumption uncertainties. Sperber *et al.* ^7^ found that the maximum shiftable load in German SFH is 57 GW *el*. Weiß *et al.* ^8^ focused on the peak shaving potential in the Austrian building stock through thermal inertia. They concluded that 50% of the heating peak loads could be shifted to off-peak hours for buildings built after 1980 by turning off the heating system until indoor temperature is reduced from 22°C to 19°C in a typical winter week. Gabrielle Masy *et al.* ^16^ found that 3–14% of the heating load can be shifted by using thermal inertia in a single, well-insulated building. While the heat transfer rate between the interior and the thermal mass is low, the amount of energy stored is still impressive. In this regard, Henryk Wolisz ^17^ suggests that by pre-heating a building for two hours, the heating demand for the following four hours can be reduced by 20% although the simulated building is made out of bricks and has no insulation. Floor heating and direct thermal activation are more effective in shifting loads with thermal inertia, however HP peak loads can still be significantly reduced with a radiator heating system and the proper control strategy ^18^.

In this work, we dive deeper into the effect of a variable electricity price for buildings operated with a HP. Among different variable electricity price mechanisms ( *e.g.* critical peak pricing, day/night tariffs, or real-time pricing), we focus on the RTP for two reasons: (1) some real-time pricing schemes for residential consumers are already established in Austria; (2) it is a straightforward way to incentivize residents to shift their demand and has been proven to be more effective than other pricing schemes ^4^.

## Methodology

### Technology operation model of individual households

We developed an hourly technology operation model for individual households to model the response of households’ energy consumption to variable electricity prices. The model calculates the operation of technologies throughout a whole year (8760 hours) in two modes: 1) benchmark mode, in which the energy demand of the building is met to keep a certain comfort level, and 2) optimization mode, in which a SEMS minimizes the operation cost of the household while keeping the same comfort level.

**Figure 1.**
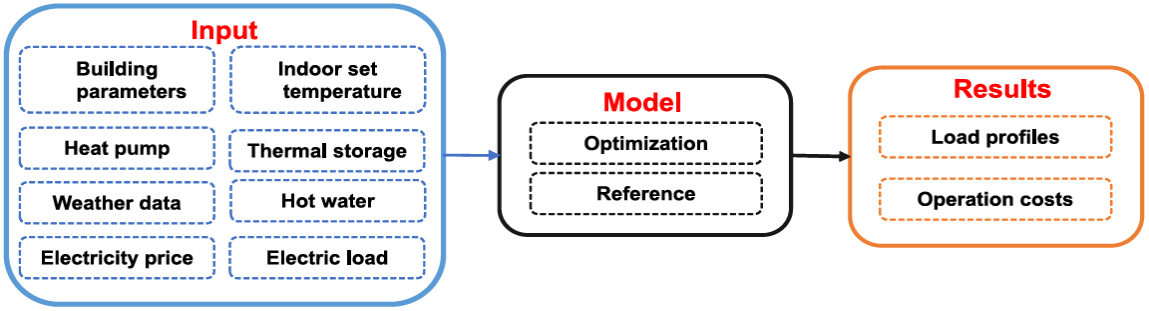
Overall structure of the model.

Figure 1 presents the model’s general set up - input and output. Further details can be found in 19 ^1^.

The relevant parts of the model for this paper are introduced as follows.

**Figure 2.**
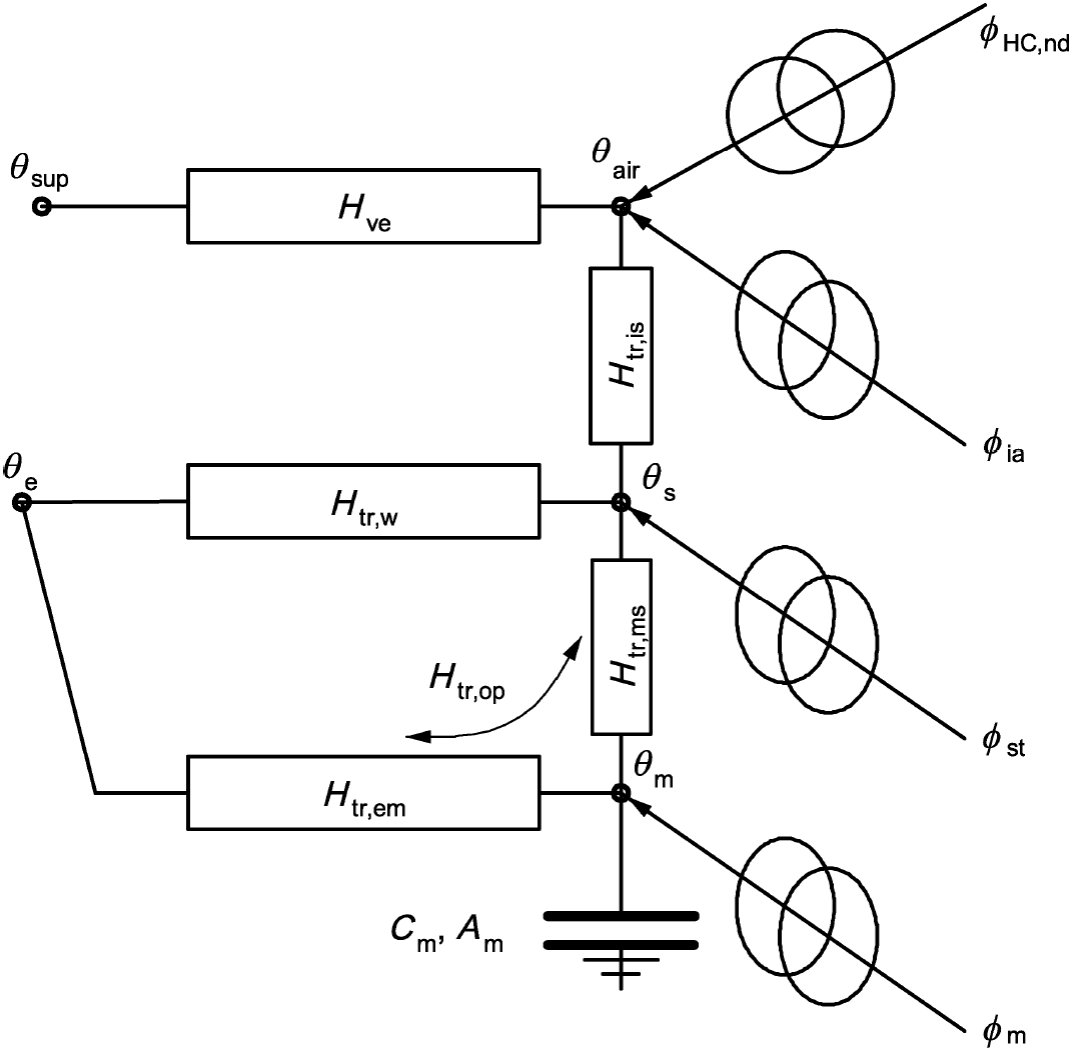
5R1C circuit representation.

The necessary heating demand is calculated with a simplified five resistance one capacity (5R1C shown in Figure 2) approach following the DIN ISO 13790. This norm is outdated and has been replaced by the DIN ISO 52016. But the computational effort to implement the DIN ISO 52016 into the optimization framework is much higher. Both modeling approaches have been compared in 21. And the 5R1C approach has been tested against results from Energy Plus, Invert/EELab and the VDI 6007 ^22– 24^. They show that the heating demand calculated by the 5R1C model is reasonably accurate, whereas cooling demand is strongly overestimated.

Figure 2 shows the circuit representation of the 5R1C model described in the DIN ISO 13790. The relation between outside temperature ( *θ _outside_*), indoor temperature ( *θ _air_*) and heating demand ( *ϕHC*) is provided by the following equations. A detailed description of the calculation methodology can be found in the DIN ISO 13790. In the following equations, *ϕ* represents any kind of heat flow in *W* and *θ* is used to describe temperatures in ° *C*. Equation 1 describes the relation between the indoor temperature ( $\theta_{air}^{t}$ ) and the provided heating energy ( $\phi_{HC}^{t}$ ).


$$\;\theta_{air}^{t}=\frac{H_{is}\times \theta_{s}^{t}+H_{ve}\times \theta_{sup}^{t}+\phi_{ia}+\phi_{HC}^{t}}{H_{is}+H_{ve}}\;(1)$$

*θ _sup_* describes the air temperature from the ventilation system. If no ventilation system with heat exchangers is adopted *θ _sup_* is set to *θ _outside_*. In this study ventilation systems are neglected.


$$\;\phi_{ia}=0.5\times \phi_{int}\;(2)$$

*ϕ _int_* describe the internal heat gains in *W*/ *m* ^2^. For simplification the internal gains ( *ϕ _int_*) are kept constant with 3.4 *W*/ *m* ^2^, although in reality they can change every hour.

The node temperature *θ _s_* is calculated through following equation:


$$\;\theta_{s}^{t}=\frac{H_{ms}\times \theta_{m_{avg}}^{t}+\phi_{st}^{t}+H_{w}\times \theta_{outside}^{t}+H_{tr1}\times (\theta_{sup}^{t}+\frac{\phi_{ia}+\phi_{HC}^{t}}{H_{ve}})}{H_{ms}+H_{w}+H_{tr1}}\;(3)$$

The average temperature of the thermal mass ( $\theta_{m_{avg}}^{t}$ ) in each timestep is calculated as follows:


$$\;\theta_{m_{avg}}^{t}=\frac{\theta_{m}^{t}+\theta_{m}^{t-1}}{2}\;(4)$$

$\theta_{m}^{t}$ and $\theta_{m}^{t-1}$ represent the temperature of the thermal mass in the current and in the previous timestep and have to be calculated for each timestep individually:


$$\;\theta_{m}^{t}=\frac{\theta_{m}^{t-1}\times (\frac{C_{m}}{3600}-0.5\times (H_{tr3}+H_{em}))+\phi_{m_{tot}}^{t}}{\frac{C_{m}}{3600}+0.5\times (H_{tr3}+H_{em})}\;(5)$$

with


$$\phi_{m_{tot}}^{t}=\phi_{m}^{t}+H_{em}\times \theta_{outside}^{t}+H_{tr3}\times (\phi_{st}^{t}+H_{w}\times \theta_{outside}^{t}+\frac{H_{tr1}}{H_{tr2}}\times (\frac{\phi_{ia}+\phi_{HC}^{t}}{H_{ve}}+\theta_{sup}^{t}))\;(6)$$

$$\;\phi_{st}^{t}=(1-\frac{A_{m}}{A_{t}}-\frac{H_{w}}{9.1\times A_{t}})\times (0.5\times \phi_{int}+\phi_{sol}^{t})\;(7)$$

with *ϕ _sol_* describing the solar gains.


$$\;\phi_{m}^{t}=\frac{A_{m}}{A_{t}}\times (0.5\times \phi_{int}+\phi_{sol}^{t})\;(8)$$

Every parameter described with an *H* represents a transmission heat transfer coefficient and is given in *W*/ *K*. All building specific parameters are described in the following equations:


$$\;H_{tr1}=\frac{1}{1/H_{ve}+1/H_{is}}\;(9)$$

with *H _ve_* being the ventilation transfer coefficient.


$$\;H_{tr2}=H_{tr1}+H_{w}\;(10)$$

$$\;H_{tr3}=\frac{1}{1/H_{tr2}+1/H_{ms}}\;(11)$$

$$\;H_{is}=h_{is}\times A_{tot}\;(12)$$

with *h _is_* being the heat transfer coefficient between the air node *θ _air_* and the surface node *θ _s_* which equals to 3.45 *W*/ *m* ^2^ *K*. *A _tot_* denotes the total surface area of the building in *m* ^2^.


$$\;A_{tot}={\text{Λ}}_{at}\times A_{f}\;(13)$$

where *A _f_* is the effectively used floor area in *m* ^2^ and Λ *_at_* represents the dimensionless ratio between the surface area of all surfaces that face into the space and the effective area. Λ *_at_* is set to 4.5.


$$\;H_{em}=1/(\frac{1}{H_{op}}-\frac{1}{H_{ms}})\;(14)$$

*H _ms_* is provided in *W*/ *K*:


$$\;H_{ms}=h_{ms}+A_{m}\;(15)$$

*h _ms_* denotes the heat transfer coefficient between the nodes *m* and *s* and is fixed with 9.1 *W*/ *m* ^2^ *K*.

*A _m_* is the effective mass-related area in *m* ^2^ and is calculated with following formula:


$$\;A_{m}=\frac{C_{m}^{2}}{\sum \left(A_{j}\times k_{j}^{2}\right)}\;(16)$$

*A _j_* represents the surface area of the building element *j* in *m* ^2^ and *k _j_* represents the specific thermal capacity of a building element *j* in *J*/ *m* ^2^ *K*. *C _m_* is provided in *J*/ *K* and denotes the total thermal capacity of the building mass:


$$\;C_{m}=\sum \left(k_{j}\times A_{j}\right)\;(17)$$

$$\;H_{op}=H_{D}+H_{g}+H_{U}+H_{A}\;(18)$$

*H _D_*, *H _g_*, *H _U_* and *H _A_* represent the transmission heat transfer coefficients for direct heat transmission to the external environment ( *H _D_*), for the steady-state heat transmission to the ground ( *H _g_*), through unconditioned rooms ( *H _U_*) and to adjacent buildings ( *H _A_*). The calculation of these four parameters is described in the DIN ISO 13789. In the following equation *H _X_* stands for either *H _D_*, *H _g_*, *H _U_*, *H _A_*:


$$\;H_{X}=b_{tr,x}\times (\sum_{i}A_{i}\times U_{i}+\sum_{k}l_{k}\times \psi_{k}+\sum_{j}\chi_{j})\;(19)$$

with *U _i_* being the heat transfer coefficient in *W*/ *m* ^2^ *K* for the respective building element *i*.

*ψ*... linear heat transfer coefficient for a thermal bridge

*l*... length of a thermal bridge

*χ*... heat transfer coefficient for a punctual thermal bridge

*b _tr,x_*... adjustment factor if the temperature if the temperature on the other side of the component is not equal to the temperature of the external environment

The total ventilation heat transfer coefficient ( *H _ve_*) is calculated as follows:


$$\;H_{ve}=\rho_{a}\times c_{a}\times (\sum_{k}b_{ve,k}\times q_{ve,k})\;(20)$$

*ρ _a_* × *c _a_*... volume-related heat storage capacity of the air in *J*/ *m* ^3^ *K*

*q _ve_*... the time-averaged air volume flow

*b _ve_*... the temperature adjustment factor for airflow element k. If the supply air temperature is not equal to the temperature of the external environment, *b _ve_* ≠ 1.

Parameters for each building are shown in Table 1. Complete data on the buildings including window areas are provided in 25 in the ”Variable_Price_Paper.sqlite” file under the table name ”OperationScenario_Component_Building”.

**Table 1.** Building IDs with 5R1C parameters ^11^.

ID	Name	Generation of renovation	Age class	*A _f_* (m ^2^)	*H _ve_*	*H _op_*	*H _tr,w_*	*C _m_* (mio. J/K)
1	SFH A mon	-	1890–1918	129	36	229	101	53
2		1				242	87	
3		2				250	78	
4		3				198	76	
5	SFH A	-	1890–1918	129	36	227	101	53
6		1				241	87	
7		2				250	78	
8		3				164	67	
9	SFH B mon	-	1919–1944	136	38	290	116	46
10		1				306	100	
11		2				316	89	
12		3				206	78	
13	SFH B	-	1919–1944	136	38	288	117	46
14		1				305	100	
15		2				316	89	
16		3				189	74	
17	SFH C	-	1945–1960	144	41	278	116	31
18		1				295	99	
19		2				304	89	
20		3				183	70	
21	SFH D	-	1961–1970	154	44	193	95	33
22		1				206	82	
23		2				212	77	
24		3				150	68	
25	SFH E	-	1971–1980	163	46	204	101	35
26		1				214	90	
27		2				226	79	
28		3				159	71	
29	SFH F	-	1981–1990	166	46	141	79	35
30		1				149	71	
31		2				142	69	
32	SFH G	-	1991–2000	170	48	120	71	31
33		1				122	68	
34	SFH H	-	2001–2008	170	48	90	59	19
35	SFH I	-	2009–2012	170	48	87	62	19
36	SFH dh	-	2012–2019	170	48	59	47	19

Space heating and hot water demand are met by an HP where the coefficient of performance (COP) is dependent on the supply and the source temperature:


$$\;COP_{t}=\eta *\frac{T_{t}^{supply}}{T_{t}^{supply}-T_{t}^{source}}\;(21)$$

*η* represents a constant efficiency factor and is set to 0.35 for air source HP and 0.4 for ground source HP ^2^. For space heating, an average supply temperature ( *T ^supply^*) of 35°C is assumed, and for domestic hot water, the supply temperature is 55 °C. If the HP charges a thermal storage, the supply temperature rises by 10 °C. Indoor temperature is set to be within 20°C and 23°C. For the air source HP the source temperature ( *T ^source^*) is the outside temperature and assumed to be a constant 10°C for the ground source. The maximal electric capacity of the HP is determined by calculating the necessary thermal power of the building and COP at design conditions. In Austria, design outside temperature is around -12°C ^26^.

Austria’s outside temperature and radiation profile is generated by weighting the profiles for every NUTS3 region in Austria by their respective residential floor area and taking the average. The data is downloaded from PV GIS ^27^ for 2019.

To determine the influence of thermal storage devices on a building to shift electric loads, we simulate a SFH once without any thermal storage device and once with a buffer storage for heating and a tank for domestic hot water (DHW). The size of the DHW storage was chosen to be 400 l, and the buffer storage for heating has a volume of 750 l. Following assumptions were made regarding the hot water storage to keep the model linear:

- The temperature inside the tank is homogenous.
- The temperature surrounding ( *T _tank_surrounding*) the tank is constant at 20°C.
- Thermodynamic properties of the water (volume, heat capacity ( *c _water_* in J/kgK) and pressure) are constant.

The following equations represent the physical limitations of the hot water tanks:


$$\;Q_{t}^{tank}=m_{water}*c_{water}*(T_{1}^{tank}-T_{min}^{tank})\;(22)$$

$$\;T_{0}^{tank}=T_{min}^{tank}\;(23)$$

$$\;28{}^{\circ}\leq T_{t}^{tank}\leq 45{}^{\circ},65{}^{\circ}\;(24)$$

$$\;Q_{loss}^{tank}=\text{Λ}*A_{tank}*(T_{t}^{tank}-T_{surrounding}^{tank})\;(25)$$

$Q_{t}^{tank}$ denotes the energy stored in a tank (J) at a particular hour with *m _water_* (kg) being the mass of the water and $T_{t}^{tank}$ the current temperature inside the tank. $T_{min}^{tank}$ is the minimum temperature inside the tank and was chosen to be 28°C. $T_{0}^{tank}$ is the temperature inside the tank at the start of the simulation. The maximum temperature of the buffer heating storage is 45°C and 65°C for the DHW storage. Both storages have the same insulation with a heat transfer coefficient (Λ) of 0.2 W/m2. The buffer storage and the DHW tank have an Area ( *A _tank_*) of 4.6 m2 and 3 m2, respectively. $Q_{loss}^{tank}$ describes the thermal losses of the storage.

In the further course, we simulate multiple representative buildings from the Austrian SFH building stock and aggregate the results to the national level.

An optimization was developed in Pyomo ^28^ and solved by Gurobi ^3^ to minimize the household energy cost. The objective function of the model is to minimize the operation costs over one year on an hourly basis:


$$\;min\;Cost=\sum_{t=1}^{8760}P_{t}*E_{t}\;(26)$$

With *P _t_* being the electricity price and *E _t_* the amount of electricity bought from the grid at a certain hour. The electricity consumption is composed of fixed and variable components. Fixed components of the electricity consumption are internal loads from appliances and lighting as well as the electricity demand of the HP for hot water generation in case there is no DHW storage. The electricity consumption profile is a synthetic load profile for a single household ^4^. The hot water demand consumption rate is assumed to be 90 l per day ^29^ with an initial temperature of 10°C and a supply temperature of 55°C. The hourly demand profile is taken from the Hotmaps project ^30^.

The variable electric load consists of the electricity demand from the HP. The SEMS can increase the heating demand in a specific timeframe by pre-heating the building and reduce it in the following hours. Additionally, if the building is equipped with thermal storages, they can be charged and discharged at any time. Thus, the SEMS can shift electricity demand for heating even more effectively.

Similar to 4 shifted energy ( *E _shifted_*) in this study was defined as the difference between the electric power consumption profiles with and without DR:


$$\;E_{shifted}=\int P_{t}^{Benchmark}-P_{t}^{SEMS}\;dt\;\;if\;\;P_{t}^{Benchmark}>P_{t}^{SEMS}\;(27)$$

$P_{t}^{benchmark}$ denotes the electric load from the grid in the benchmark case and $P_{t}^{SEMS}$ the load with a SEMS installed. If $P_{t}^{SEMS}$ is higher than $P_{t}^{Benchmark}$ the building is being pre-heated or storages are charged. Likewise the energy is shifted whenever $P_{t}^{Benchmark}$ is higher than $P_{t}^{SEMS}$ . Naturally the shifted energy is is lower than the energy used to charge storages and building mass due to thermal losses.

The benchmark model cannot shift any loads. Heating demand is calculated based on the same 5R1C approach with the assumption that the air temperature inside the building should not drop below 20°C. The HP then provides the needed heating power with the corresponding COP. As there is no non-smart logic on when to fill and discharge a thermal storage based on the variable price, the benchmark model does not adapt thermal storages.

**Figure 3.**
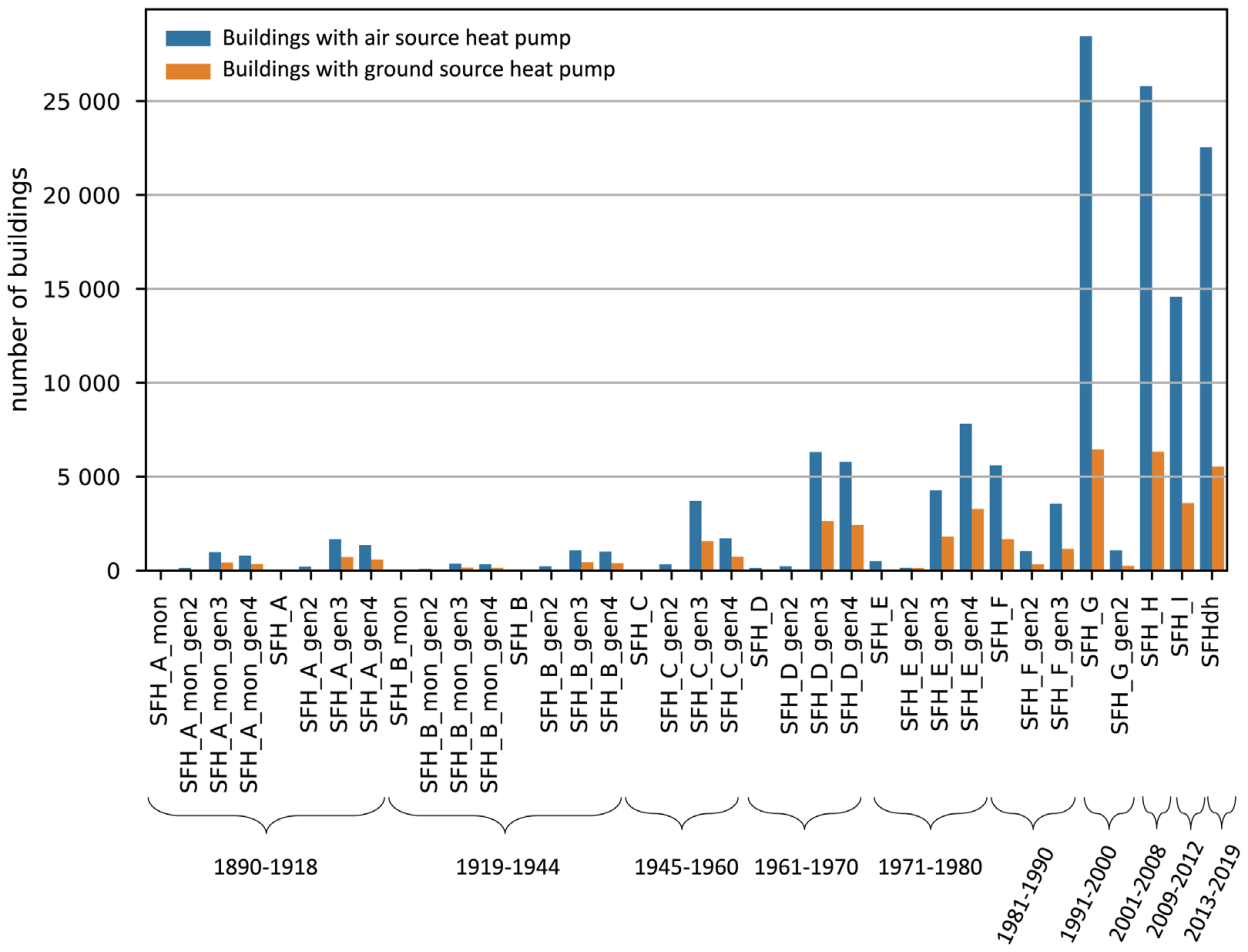
Number of buildings with HPs ins the Austrian SFH building stock ^11^.

To capture the possible impact of price incentivized load shifting of the Austrian SFH building stock, we coupled our model to the INVERT/EELab building stock model ^11^. In this database, the SFH building stock in Austria is represented by 36 building categories. Each category represents a typical SFH built in a certain time period and its overall thermal insulation properties. The different representative buildings and the installed number of HP in each category are presented in Figure 3. More information about the buildings is provided in Table 1. The appendix ”mon” indicates that the buildings are under protection. Gen2, gen3, and gen4 indicate that these buildings have undergone a certain renovation, with higher numbers representing more recent renovations. We can see that most HPs have been installed in modern buildings. Overall Austria has around 1.55 million SFHs out of which 180 000 have a HP installed ^5^.

### Electricity price scenarios

The electricity price is the driving factor of the energy cost minimization problem. Four different electricity profiles are investigated in this study to establish a relation between the volatility of the electricity price and the shifted load by the building. The first profile is Austria’s actual day-ahead electricity spot price 2021 from ENTSO-E ^6^. A hypothetical fixed grid fee plus taxes of 20 cents/kWh are added in order to derive the related electricity retail price.

In future decarbonized electricity systems, a higher price variance on electricity spot markets is expected for systems with very high shares of renewable generation ^3^. This is because of more hours with spot prices close to zero due to very high shares of renewable generation with low variable generation costs. In times of low renewable generation and high load on the other side, flexibility options at higher costs like dispatchable generation units, storage discharge, or import set the marginal price, which results in relatively high spot prices. The Austrian electricity system for 2030 is modeled with the bottom-up energy system model Balmorel ^9, 10^ to reflect such a decarbonized power system with higher price variance. The aim is to reflect various electricity market settings where different price patterns occur. We model three price scenarios to analyze sensitivities differing in the assumed CO _2_ price. The CO _2_ price influences the high-price hours when gas-fired units in this system define marginal costs. However, this could be any other flexibility option at a high price level in a different market setting. The hypothetical fixed grid fee plus taxes of 20cents/kWh are also added in order to derive the retail electricity price.

In Balmorel we model the Austrian electricity and district heat system as well as the neighboring countries Czech Republic, Germany, Hungary, Italy, and Slovenia to capture export and import dynamics. Installed generation capacities, national electricity demand, and net transfer capacities are based on the Ten-Year Network Development Plan (TYNDP) 2020 National Trends scenario ^7^. For Austria, the assumptions are based on the national energy and climate plan WAM scenario ^8^ and are refined to reflect the 100% renewable target (nationwide in annual balance terms) until 2030. That means that Austria meets its goals defined in the renewable expansion act ^31^ and becomes a net exporter of electricity. The installed capacities in Austria for these simulations are depicted in Table 3 in the Annex. All electricity generated by natural gas is exported (on annual balance). The model with input parameters has been tested and validated in 32, 33.

**Figure 4.**
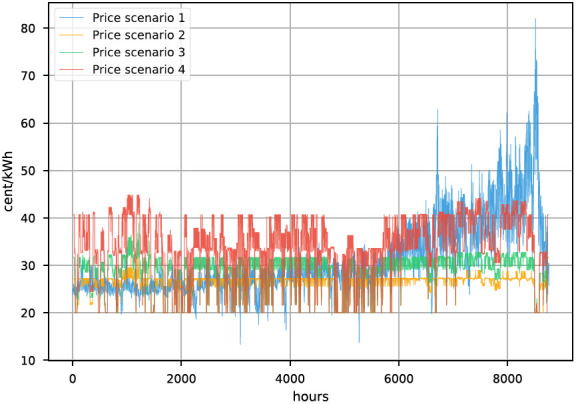
Electricity prices.

The resulting mean plus standard deviation for all price profiles are provided in Table 2. With an increasing CO _2_ price, the price profile’s mean value and volatility increase. All prices are depicted in Figure 4.

**Table 2.** Mean and standard deviation of the price scenarios and CO _2_ price as well as the number of local maxima in the profile.

Price scenarios	mean (cent/kWh)	CO2-price (€/tCO _2_)	Number of local maxima
Price 1	30.69 ± 7.76	-	1225
Price 2	26.05 ± 2.27	53	621
Price 3	28.87 ± 3.52	106	646
Price 4	34.11 ± 6.35	212	618

**Figure 5.**
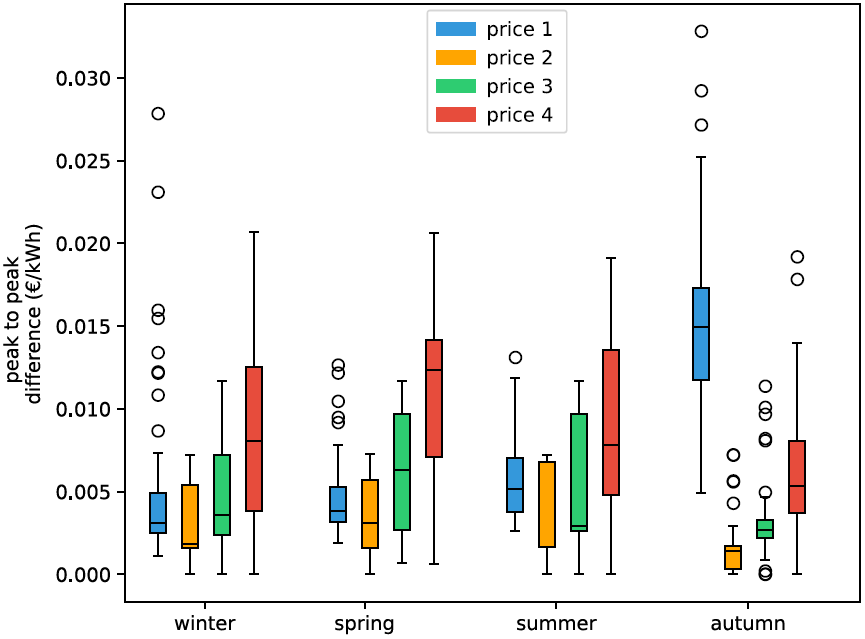
Box plots of seasonal effect in electricity price based on daily peak-to-peak amplitude difference. Box plots show the median (horizontal line) and the interquartile range (IQR) (box outline). The whiskers extend from the hinge to the highest and lowest values within 1.5 × IQR of the hinge, and the points represent the outliers.

In Figure 5 the daily peak-to-peak amplitude difference of all four prices is visualized. The peak-to-peak difference in the day ahead price from 2021 increases drastically throughout the year. In the modelled prices for 2030 incisive events, like energy shortages, are not considered, thus the prices are not fluctuating that much throughout the year.

Another significant difference between the 2021 real time price and the simulated prices is the frequency of change. It is visible in Figure 4 that price 1 is changing more frequently than the others. Table 2 shows the number of local maxima in the respective price profiles. The price from 2021 has about twice as many peaks as the prices from 2030 (price 2, 3 and 4). In this work, we focus on volatility in terms of price spread. Structural price volatility in the form of higher frequency can also significantly impact the potential of load shifting. Studies showed that HPs could participate in the intra-day and even the frequency regulation market ^34, 35^. But in order to participate in the regulation market they need to be pooled in order to reach the necessary capacity ^36^. In this study we do not consider participation of residential HPs other than the day ahead market. Thus price frequencies are limited to hourly changes. Although the thermal inertia of a building is slow, high frequency of price change generally results in higher cost savings for HPs if they react to the price signal.

## Results and discussion

The data for all results describe in the following chapters is available at 25.

### Results of individual households

In this section, the effects of different RTP schemes on an individual household optimization are analyzed. The single building chosen to investigate the single load profiles is a typical modern SFH in the Austrian building stock, built after 2010. The building has a floor area of 170 m ^2^ and a useful energy demand for space heating of 45 W/m ^2^. The thermal capacity of this building is low and the insulation level high compared to older buildings in the building stock. Then the same building is simulated with a 400l DHW storage and a 750l space heating buffer storage to show the difference in load shifting potential by implementing thermal storages.

**Figure 6.**
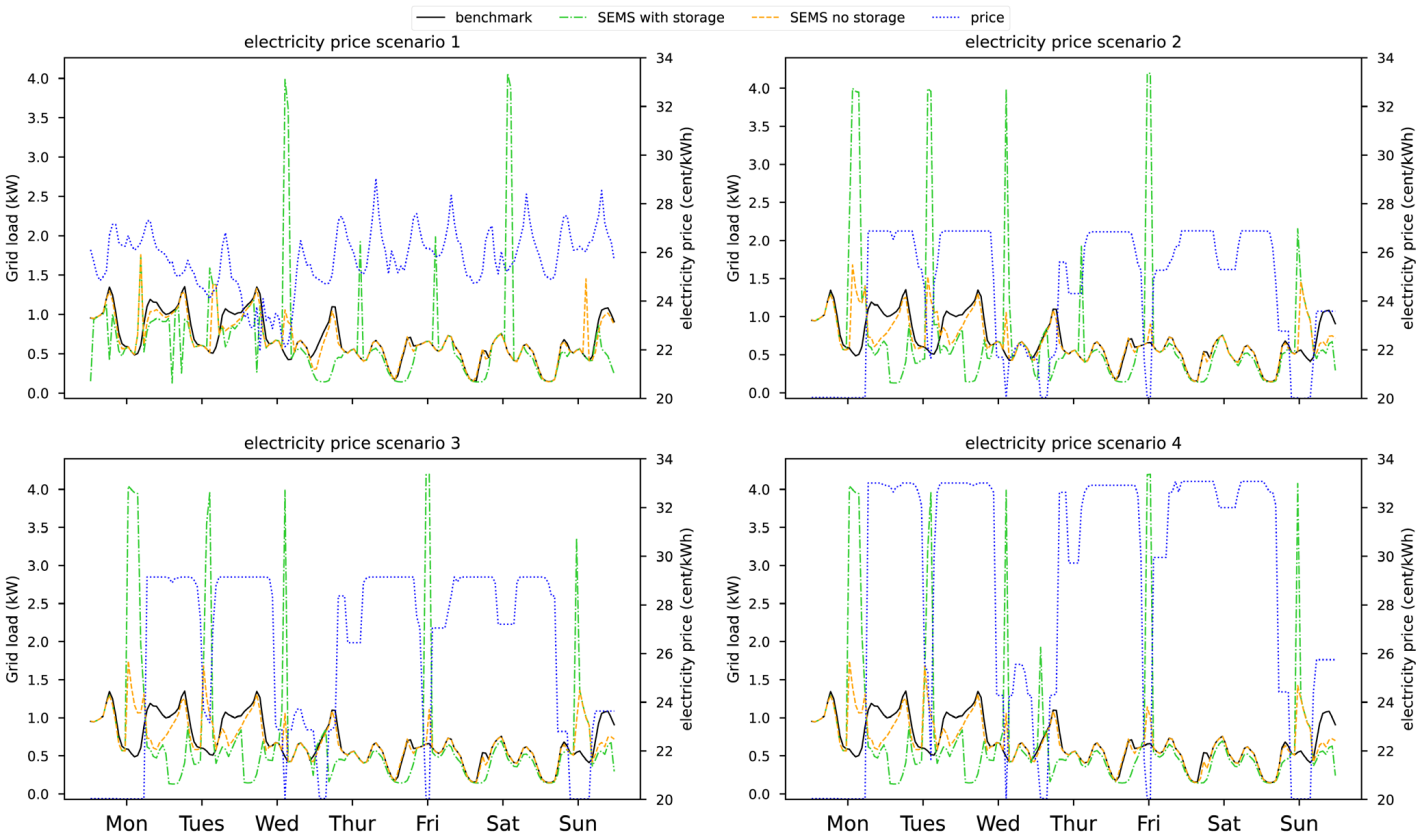
Electricity bought from the grid with and without SEMS in the 12 ^th^ week of the year. The electricity price is visualized on the right-hand axis.

In Figure 6 the grid electricity demand for a household in the 12 ^th^ week is visualized. The three load profiles represent the load if no load shifting is happening (benchmark), load shifting without any thermal storage (SEMS no storage) and load shifting with a DHW and heating buffer storage (SEMS with storage). On the right-hand y-axis, we see the electricity price profile. We chose the 12 ^th^ week because during this period price 1 is similar to prices in the years prior to 2021.

Looking at the price scenarios 2, 3, and 4 we see that the higher price difference only increases the incentive to shift loads marginally when thermal storages are available. A change of 6 cent/kWh is enough to charge the thermal storages with the HP. Without thermal storage the peak loads during low energy price times, are limited by the maximum indoor set temperature. With higher price volatility the building is pre-heated to a greater extend if possible.

**Figure 7.**
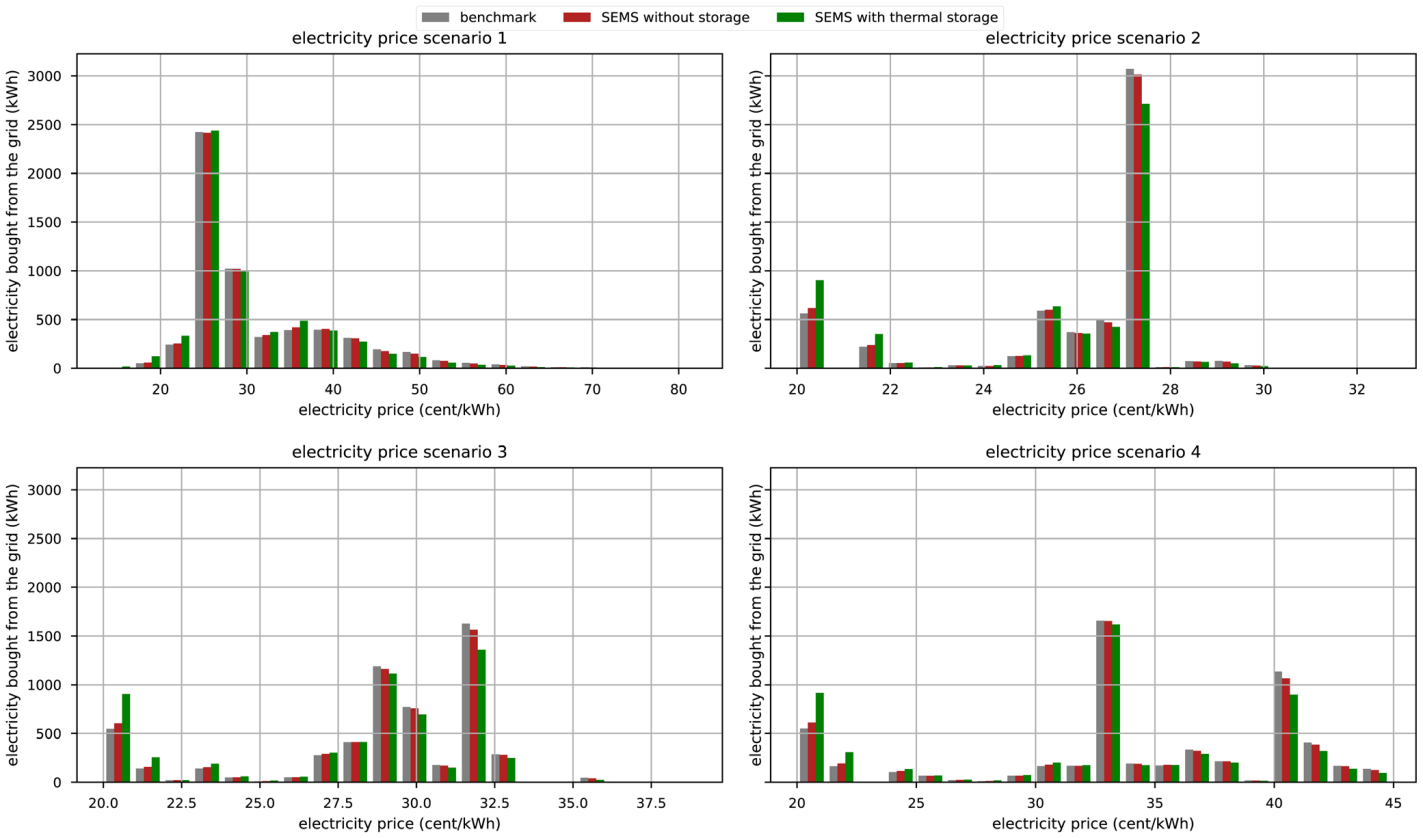
Electricity demand at certain prices in the benchmark case and with the implementation of SEMS. ”With thermal storage” indicates that the house has a 750 l buffer storage and a 400 l DHW storage.

Figure 7 shows the amount of electricity bought at a specific price. The benchmark case is directly correlated to the occurrence of the electricity price. But when the household is optimized, electricity is bought at lower prices and reduced at higher prices. In price scenarios 2,3, and 4 the amount of electricity purchased from the grid when the price is at its lowest almost doubles when the household has the thermal storages implemented. Even without thermal storage, the increase in electricity consumption at lower prices and the decrease at higher prices are visible.

**Figure 8.**
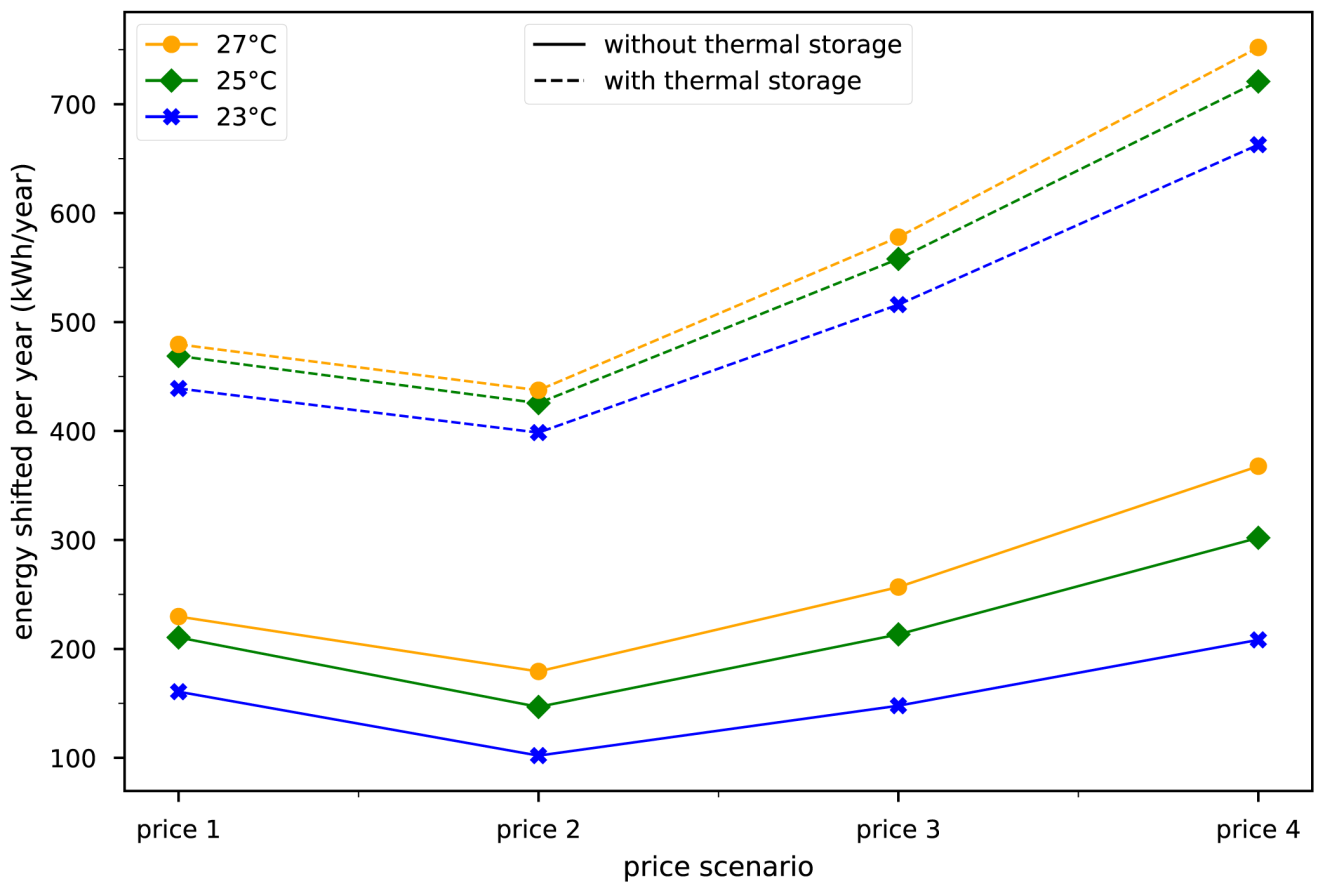
Energy shifted over one year in the building with no thermal storage and the building with DHW storage and buffer storage. The temperatures represent the maximum allowed indoor temperatures during winter.

The maximum average indoor set temperature was varied between 23, 25, and 27°C during winter to investigate the sensitivity of the allowed indoor set temperature for pre-heating. Figure 8 shows that higher electricity price volatility increases the incentive to shift energy by pre-heating the building. Comparing the price scenarios 1 to 2,3 and 4, we see that price 1 creates a greater incentive to shift load than price 2 and 3 without the thermal storage. This is due to the nature of the price profiles. Price 1 is changing much more frequently than the other 3 prices. But with the implementation of thermal storages shifted demand is much higher in the price scenarios 3 and 4 and even in price 2 the shifted energy is almost as high as in price 1. The thermal storages compensate for the lower frequency of price changes by storing much more energy in a short period of time. The correlation between the allowed indoor temperature bandwidth and the energy shifted by the optimization is not linear because of the increase in thermal losses with higher indoor temperatures.

If the building has installed thermal storages, the shifted energy rises to around 450 kWh/year in the first price scenario. This result is in line with 4 where a building with 310 m2 floor area, 2000 l buffer storage, and 400 l DHW storage shifts a maximum of 1370 kWh of electric energy. The allowed indoor temperature is still impacting the total amount of shifted load even though the load shifting is done predominantly by the storages. The amount of energy that is shifted with the implementation of thermal storages is by a multitude higher in this case. The absolute amount of shifted energy through thermal storages is almost independent of the building type. Buildings with higher thermal mass and higher energy need, can shift more electricity through the thermal mass. However thermal storages are always prioritized for load shifting because the losses are smaller. Thus any price change which would incentivise a house to pre-heat will give an even higher incentive to shift load *via* the thermal storage.

**Figure 9.**
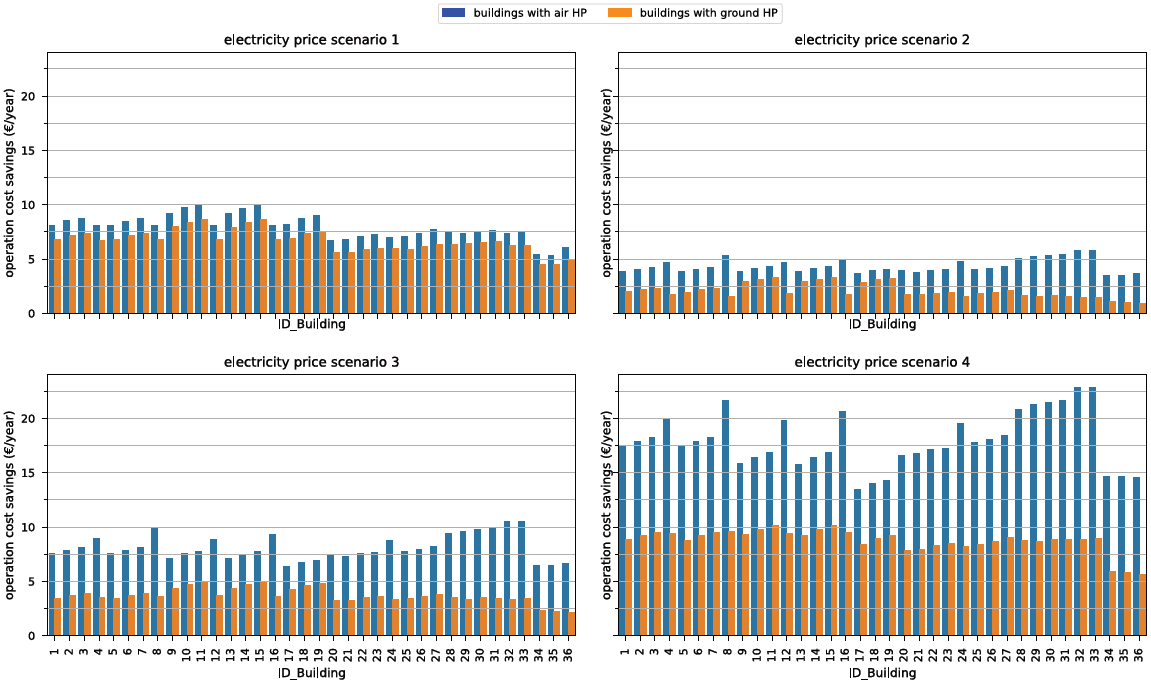
Operation cost savings per building type and HP type in all four electricity price scenarios.

In Figure 9, the operation cost savings for each building type are visualized without any thermal storage. With a higher volatility in price change, the economic optimization of HP becomes more lucrative. The optimization is less lucrative for modern buildings. These buildings are characterized by lower thermal mass and a higher degree of insulation. Thus, they have a much lower heat- and electricity demand. The same reason accounts for the difference between air source and ground source HP since the ground source HP is more efficient. Because of the high volatility in the 2021 price profile at the end of the year, the implementation of a SEMS is more lucrative in the buildings compared to the second price profile. A sensitivity analysis showed, that with prices prior to 2021, economic gains through the SEMS were always lower than in the second price scenario. The operation cost reductions are generally between EUR 2 and EUR 23 depending on the price profile and the energy demand of the house. If these buildings adapt a 750l heating buffer and a 400l DHW storage, the cost reductions would rise between EUR 5 and EUR 95. Therefore, implementing a SEMS into buildings that would use only the thermal storage for demand shifting must be relatively cheap to yield return of investments after a few years. Without additional monetary incentives, homeowners are unlikely to tap into the potential of load shifting through the thermal mass alone. This finding is in accordance with other studies that suggest that the additional investments by individual buildings will not be compensated by participating in DR ^37^.

### Upscaling to the building stock

In this section, the potential load shift for the whole SFH building stock in Austria is presented in dependence on the four electricity price signals. The used methodology however can be applied to any other European country.

**Figure 10.**
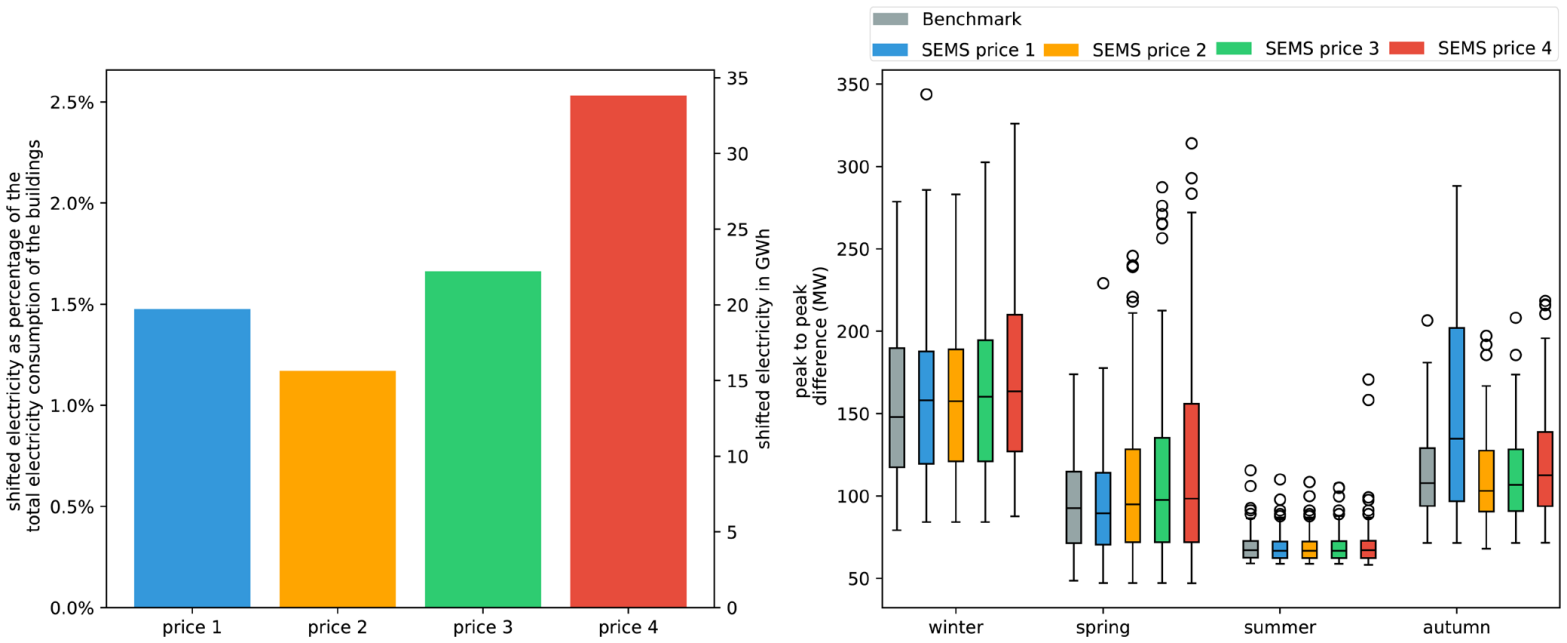
Left: Amount of electricity shifted throughout the year based on the different price scenarios. Right: Box plots of seasonal effect in grid demand, based on daily peak-to-peak amplitude difference. Box plots show the median (horizontal line) and the interquartile range (IQR) (box outline). The whiskers extend from the hinge to the highest and lowest values within 1.5 × IQR of the hinge, and the points represent the outliers.

The total amount of electricity shifted over one year in each price scenario is visualized in Figure 10 on the left side. In this case only the thermal mass of the buildings is used for load shifting. The potential of the building stock to shift load *via* HPs is around 20 GWh when considering the price from 2021. This corresponds to approximately 1.5% of the electricity consumed by these households within the same time period. The increasing volatility in price scenarios 2, 3 and 4 leads to a significant increase in electricity being shifted showing that variable price profiles can give an increasing incentive to shift loads. On the right hand side of Figure 10 the maximum daily peak-to-peak difference of the aggregated load profiles of SFH with HP in the Austrian building stock is visualized. The benchmark case represents the peak-to-peak demand for the case of no DR. In literature, it is often mentioned that load shifting will result in an even higher peak demand ^14^. In the resulting aggregated load profiles, this effect is only marginally visible except in autumn. The electricity price in 2021 is becoming so volatile that daily peak-to-peak loads are significantly higher than the benchmark and all the other scenarios. The peak-to-peak difference of all HP heated SFH buildings in Austria reaches a maximum of 350 MW. The peak demand in the Austrian grid is in the magnitude of 80 GW. Therefore potential of the shifting electricity through the thermal mass of SFH is small but not neglect-able.

**Figure 11.**
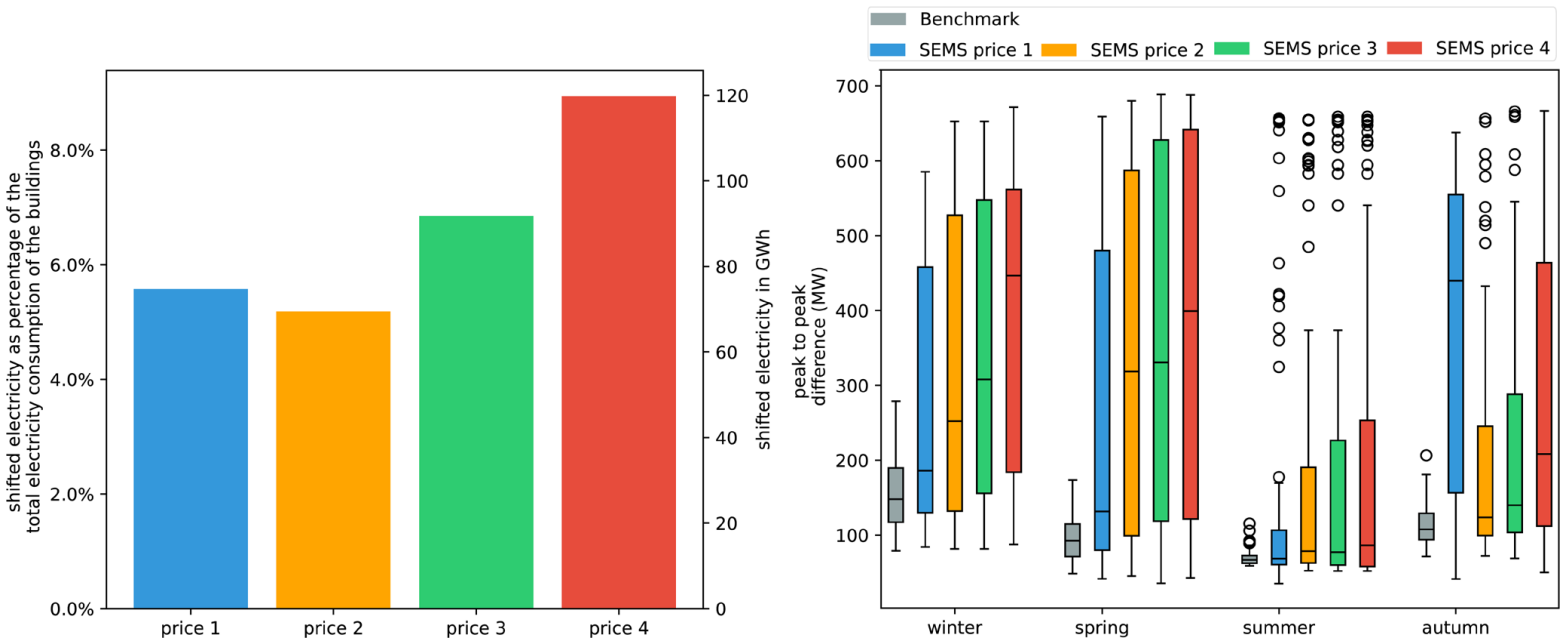
Left: Amount of electricity shifted throughout the year based on the different price scenarios with buildings having thermal storages. Right: Box plots of seasonal effect in grid demand, based on daily peak-to-peak amplitude difference. Box plots show the median (horizontal line) and the interquartile range (IQR) (box outline). The whiskers extend from the hinge to the highest and lowest values within 1.5 × IQR of the hinge, and the points represent the outliers.

As shown before, buildings with implemented storage capacities can shift a lot more electricity. To see how strongly storage capacities can influence the peak-to-peak difference on a daily basis and the overall shifted demand we generated a scenario where every single building with a HP has a 750l heating buffer storage and a 400l DHW storage. The real market penetration of hot water storages in HP operated buildings in Austria is unknown. The following results serve as a benchmark. In Figure 11 we can see that the peak-to-peak difference is more than two-folding compared to the benchmark, reaching 700 MW. Also peak demand is reached throughout the year independent of the price scenario. However the occurrence of high peak-to-peak demands increases with increasing price volatility. The total amount of electricity shifted rises to 74 GWh in price scenario 1 which is almost four times higher than without thermal storage capacity.

The results show that HPs can effectively shift electricity through the thermal mass. Peak loads increase only marginally for buildings without any storage capacity. Thermal losses that occur when pre-heating a building significantly limit the maximum power peak when optimizing such buildings’ heating demand. However, with available storages the peak-to-peak demand can more than double. This is especially relevant when HP penetration in a certain region is very high, and HPs are operated with the same strategy. This problem can be avoided by providing different price incentives for users. It is therefore crucial that the price signal reflects electricity scarcity in such a system. Overall the results show that there is significant potential for DR in the residential building stock. This potential can increase even further in the future depending on decarbonisation ambitions and energy scarcity. Furthermore, the potential for the thermal mass is bigger if temporarily cooling down buildings to a certain temperature ( *e.g.* 18°C) is considered as well.

## Conclusion

The findings in this paper indicate that through the use of RTP in HP operated SFH, large amounts of the electricity can be shifted by the use of thermal mass. An increase in the volatility of such RTP naturally increases the incentive to shift demand. In the year 2021, SFHs in Austria with HPs would shift 19.7 GWh of electricity to lower operating costs if an SEMS was installed without considering any kind of thermal or electrical storages. Increasing the volatility of price changes increases the amount of shifted electricity. However, the fiscal incentive to shift demand is unlikely to trigger investments for additional equipment to participate in DR. The installation costs of such an SEMS would strongly impact the decision to implement it. For a single building yearly savings are between EUR 2 and EUR 23 (without thermal storage) and EUR 5 and EUR 95 (with thermal storage) depending on the RTP and the building parameters. Further, we showed that the economic optimization of current buildings in the building stock is slightly more lucrative for buildings with higher energy demand. Comparing the peak-to-peak difference of the resulting profiles in the Austrian SFH building stock with HPs, higher peak loads can be expected when optimizing the HP based on a price profile. However, the effect is limited for buildings without storage devices. Further, the resulting peak loads of individual buildings will have to be compared to the overall electricity demand in the region.

One limitation of this paper is the neglect of changing prices due to load shifting by the buildings. A large penetration of HP-operated buildings that shift loads could lead to a self-cannibalism effect. Further, the possibility of extending the thermal comfort within the buildings down to 18°C for a short period of time would generate additional load shifting potential. The real amount of heating buffer and DHW storages has not been considered for the whole building stock due to the lack of data. Therefore, our results can only serve as a benchmark to evaluate the impact of SEMS on load shifting. Another aspect that will be considered in further studies are multi family buildings and photovoltaic installations combined with HP. A photovoltaic system would decrease the electricity consumption from the grid and, at the same time, increase the profitability of a SEMS because the SEMS will increase the self-consumption rate of the photovoltaic system.

## Ethics and consent

Ethical approval and consent were not required.

## Data Availability

Zenodo: Results from the FLEX Model for the paper “Impact of variable electricity price on heat pump operated buildings” https://doi.org/10.5281/zenodo.7299360 ^25^. This project contains the following underlying data: - Variable_Price_Paper.sqlite (Contains the input data and results for the scenario without any storage capacities.) - Variable_Price_Paper_SFH_23.sqlite (Contains the input data and results for the single building with a maximum indoor set temperature of 23°C during winter.) - Variable_Price_Paper_SFH_25.sqlite (Contains the input data and results for the single building with a maximum indoor set temperature of 25°C during winter.) - Variable_Price_Paper_SFH_27.sqlite (Contains the input data and results for the single building with a maximum indoor set temperature of 27°C during winter.) - Variable_Price_Paper_TS.sqlite (Contains the data for model input and results for the scenario with the 750l heating buffer storage and the 400l DHW tank.) Data are available under the terms of the Creative Commons Attribution 4.0 International license (CC-BY 4.0).

## Footnotes

^1^The Code is openly accessible under https://github.com/H2020-newTRENDs/FLEX ^20^.

^2^ *η* was chosen to make the COP of the HP consistent with data from manufacturers: Bosh, Viessmann, Helios

^3^ Mathematical programming solver, https://www.gurobi.com/

^4^ available under: https://www.apcs.at/de/clearing/technisches-clearing/lastprofile, last visited: 17.11.2022

^5^ Statistik Austria: https://www.statistik.at/

^6^ available under: https://www.entsoe.eu/, last visited: 29.06.2022

^7^ availbale under https://tyndp.entsoe.eu/, last vistited: 29.06.2022

^8^ Umweltbundesamt, last visited: 17.11.2022

## Software availability

**Source code available from: https://github.com/H2020-newTRENDs/FLEX**

**Archived source code at time of publication: https://doi.org/10.5281/zenodo.7330631** ^20^

**License: MIT**
