# Supplementary material for: Impact of variable electricity price on heat pump operated buildings
Source: Open Res Eur. 2022 Dec 7;2:135. [Version 1] doi: 10.12688/openreseurope.15268.1 (PMC10445833; doi:10.12688/openreseurope.15268.1)
Supplement: Peer review history

# Peer review: Reviewer response for version 1

**DOI:** 10.21956/openreseurope.16510.r30467

**Published:** 2023-01-17

**Authors:** Wiebke Meesenburg

**Affiliations:** 1 Section of Thermal Energy, Department of Civil and Mechanical Engineering, Technical University of Denmark, Lyngby, Denmark

The presented paper investigates the load shift potential of single-family houses equipped with heat pumps in Austria by demand side management. The analysis is based on the Austrian single family building stock, Austrian temperature profiles and historic electricity prices as well as electricity price forecasts for the year 2030. The authors conclude that the load shift of the overall building stock without thermal storages in the buildings is small compared to the overall electricity demand in Austria but not negligible. When equipped with thermal storage for domestic hot water and space heating the buildings can shift up to twice the load compared to the case without storage. The authors discuss that widely employed demand side management based on real time pricing may lead to local congestions in the electricity grid. Which may have to be taken into account for the design of future tariff structures.

 Overall, the paper is well written. The problem formulation is clear and motivated. The research question addressed is highly topical and interesting to different stakeholders not only in Austria, but also in other countries. The method applied is appropriate. The description of the method should however be improved, by a) dividing into more subchapters and b) revision of the part Technology operation model of individual households. I suggest to revise the explanations of all variables (see detailed comments below) and to also add a nomenclature. The presented results are explained clearly and support the conclusions.

 Please, also address the following minor comments and suggestions:

Introduction:

1. In the readers guide at the end of the introduction all sections are called “section b”, please revise.

Literature review:

1. The following terms are used without being defined. I suggest to introduce them and clarify how they relate to the terms used in the current paper:
  1. *Flexibility* (page 3, regarding reference [4] Fitzpatrick *et al.*)
  2. *Dynamic time of use pricing* (Is this different to “ *real time pricing*” introduced by you?, How?, page 3).
  3. *Direct load control (page 3).*
2. Regarding reference [7] Sperber *et al.*: You write that the maximum shiftable load in German SFH is 57 GW _el_. Please, specify which number of SFH was considered and for which period of time this load may be shifted.
3. Be consistent with the use of names when referencing literature (sometimes you used only the last names, other times you used both first name and last name (e.g. Gabrielle Masy and Henryk Wolisz).

Method:

1. Figure 1. The model/figure needs to be explained in more detail. "Heat pump" and "thermal storage", "Hot water" are not input parameters. Please, clarify, which input parameters are used for these appliances. In the "model" box: Do "Optimization” and "Reference” refer to case 1) and 2) described in the text? Please, clarify.
2. Page 5 and 6: It seems to me that the following variables are not explained. Please check:
  1. θ _outside, _θ _s, _θ _e_
  2. Ф _ia_, Ф _st_
  3. H _tr1_, H _tr2_, H _tr3_, H _op_
3. Page 6: Is *effective area* and *effectively used floor area* the same?
4. Page 6: Please, add a reference for the assume heat transfer coefficient or specify how they were determined.
5. Page 8: Add a reference for NUTS3 or add an explanation.
6. Page 8. Equation (22). Shouldn’t it be *T _t_* ^tank^ instead od *T _1_* ^tank^?
7. On page 6 you used t to indicate the time step as a superscript, while on page 8 you use it as a subscript. Please, align to be consistent.
8. Page 8: Add an explanation for why 28 °C was chosen as the minimum temperature in the tank. I would assume this to be higher in practice for domestic hot water tanks (due to risk of legionella growth).
9. Page 8: Specify which Area is meant by A _tank_ and use superscript for writhing m **^2^.**
10. Page 8: You write *: Heating demand is calculated based on the same 5R1C approach with the assumption that the air temperature inside the building should not drop below 20°C. The HP then provides the needed heating power with the corresponding COP.*

It does not become entirely clear, whether the heat pump is always providing the load to keep the temperature constant at 20 °C OR does the heat pump turn on when the temperature drops to 20 °C and then heats up the room to an upper threshold temperature? Please, clarify.

1. Page 9: You mention that the electricity price is the driving factor of the energy cost minimization problem. Please, add a description of how the varying COP of the heat pump due to changes in the outdoor temperature influences the optimization.
2. Page 9: What is meant by the following sentence? Please explain in more detail: However, this could be any other flexibility option at a high price level in a different market setting.
3. Page 9: Add a spacing between value and unit (20_cents/kWh).

Results:

1. First paragraph on page 11: I think this paragraph should be moved to the method section.
2. Page 11: Please, argue why you looked into room temperatures of up to 27 °C (this seems very high) and not into lower room temperatures of possibly down to 18 °C.
3. Page 13: Economic results for SFH with and without storage. Please, expand on these results. Are these annual savings? Were the investment costs for the storages taken into account? What does "relatively cheap" mean?
4. Page 15: Correct the word “ *neglect-able”* at the end of the first paragraph.
5. Page 15: Second paragraph. You use the terms *peak demand* and *peak-to-peak demand*. Please introduce both in the context of your results and possibly explain the difference.
6. Page 15: You write: capacity. Thermal losses that occur when pre-heating a building significantly limit the maximum power peak when optimizing such buildings’ heating demand. It is not entirely clear to me what you refer to. Please, explain in more detail.

Format:

1. Past tense should be used when writing about your own present work. Present tense should be used when referring to previously published work (Gastel and Day, 2017 ^1^).

Is the study design appropriate and does the work have academic merit?

Yes

Is the work clearly and accurately presented and does it cite the current literature?

Yes

If applicable, is the statistical analysis and its interpretation appropriate?

Not applicable

Are all the source data underlying the results available to ensure full reproducibility?

Yes

Are the conclusions drawn adequately supported by the results?

Yes

Are sufficient details of methods and analysis provided to allow replication by others?

Yes

Reviewer Expertise:

Thermal energy systems, heat pumps, flexible operation of heat pumps, dynamic system modelling, integration of heat pumps with district heating

I confirm that I have read this submission and believe that I have an appropriate level of expertise to confirm that it is of an acceptable scientific standard.

## References

[1] : How to Write and Publish a Scientific Paper. *Cambridge University Press* .2017;

# Peer review: Reviewer response for version 1

**DOI:** 10.21956/openreseurope.16510.r30465

**Published:** 2022-12-12

**Authors:** Evelyn Sperber

**Affiliations:** 1 Department of Energy Systems Analysis, Institute of Networked Energy Systems, German Aerospace Center (DLR), Stuttgart, Germany

**Summary of report:**

 This paper analyzes the effects of a cost-minimized heat pump operation considering different thermal storage options and different real time price scenarios, taking the example of Austria. The authors find that large amounts of electricity can be shifted with real time pricing, especially if thermal storages are used and if the volatility of prices is high. However, cost savings for households are limited and thus unlikely to provide enough monetary incentives for participating in Demand Response.

**Overall impression:**

 Introduction:

- Your introduction has a very clear storyline and is very well-structured. The objective of your research is very clear and you did a good job explaining the context as well as the state of research.
- Your case study is limited to Austria. That is okay, but for a better interpretation of results it would be nice if you could inform the reader about the (projected) relevance of heat pumps for Austrian buildings.

Methodology:

- Generally, your methodology description is well-written: it is widely clear, thorough and also clearly states the assumptions made.
- You write: *“And the 5R1C approach has been tested against results from Energy Plus, Invert/EELab and the VDI 6007. They show that the heating demand calculated by the 5R1C model is reasonably accurate, whereas cooling demand is strongly overestimated*.“ Have you also tested the 5R1C model against Energy Plus etc. regarding the replication of thermal inertia? I think that this point is even more important than the good accuracy regarding the heating demand estimation, as it determines the load shifting potential. Literature gives some hints that 1 C might be not enough to adequately reflect the thermal inertia (see e.g. Bruno *et al.*, 2016 ^1^).
- I recommend to reconsider the representation of heat pumps in your model. The average supply temperature of 35°C seems very optimistic (very low) for old buildings that are not well insulated, as far as I understand from Figure 1. Is it possible that you vary the supply temperature according to the building type?
- I’m not sure if the COP formula (Equation 21) is correct. Is it possible that the reciprocal value of *η* should be used? Otherwise COP values below 1 are obtained (for instance if using 35°C supply temperature and 10°C source temperature).
- I found it a bit difficult to follow your objectives and partly methods considering the electricity price scenarios you investigate.
  - Could you please explain how electricity prices are derived from BALMOREL? Does the model incorporate a day-ahead market representation?
  - Is the “benchmark” heat pump operation considered in the load for BALMOREL (to ensure that price patterns somehow reflect the electricity demand of heat pumps?)
  - I find it a bit arbitrary to use 2030 electricity price scenarios without changing the heat pump market penetration compared to 2021. Why do you do so? Is there no large uptake of heat pumps foreseen in Austria for the next years (like in Germany, for example)?
  - I think it would help the reader when you state already at this point that you do not consider changing prices due to load shifting. I think this is an important limitation that should already be addressed in the methodology.
  - Maybe a small explanation regarding the price increase in scenario 1 in the course of the year would be helpful (e.g. refer to gas crisis).

Results:

- Your explanation of the results is generally very clear, and the figures you use are very illustrative. You provide interesting sensitivity analyses, e.g. regarding the maximum average indoor set temperature. It is great that you provide the data of your results!
- You write: *“However thermal storages are always prioritized for load shifting because the losses are smaller.” *Also, when having a look at Figure 8, it seems that there is no need to make use of the thermal mass of buildings as the most relevant effect stems from thermal storage. Maybe you should put this a bit into perspective, considering that households have to invest in thermal storages, while thermal mass is available for free.
- As cost savings are quite limited for households, have you considered to further increase price volatility (especially downwards) by providing time-dependent grid fees plus taxes (instead of fixing them at 20 ct/kWh). For instance, if taxes are somehow linked to electricity wholesale prices, the minimum of your prices could fall below 20 ct/kWh, and consequently the use of thermal storage could be increased. I think the load shifting effects in your study are quite small, because real time prices are generally high, and thus storage is not used so much (because use of storage triggers thermal losses that have to be paid). I would at least suggest that you discuss this a little.

Conclusion:

- Your conclusion captures the storyline of the paper, and gives a good overview of what you’ve done. It is also very good that you address important limitations.
- I only miss some conclusions regarding the load shifting potential when a thermal storage is used. Maybe you can add a sentence?

**Additional smaller remarks:**

 Introduction:

-  “ *In the benchmark mode, the operation of technologies is simulated based on specific assumptions, […]”* -> Unclear. Do you mean “inflexible”, heat demand oriented operation?
- I actually find the wording “benchmark” quite misleading. The terminus implies that this is the best case (in whatever regard). In terms of cost, however, it is “worse” than the SEMS. Maybe you reconsider the wording and instead use something like “baseline” or “reference”?  
-  “ *The remainder of this paper is structured as follows […]*” The following references seem incorrect – they all refer to “section b” which is the literature review.

Results and Discussion:

- *“The benchmark case is directly correlated to the occurrence of the electricity price.” *-> I do not understand this sentence. What do you mean by “occurrence of the electricity price”?

Footnotes:

- Footnote 2: little spelling error regarding Bo **s**ch.

Is the study design appropriate and does the work have academic merit?

Yes

Is the work clearly and accurately presented and does it cite the current literature?

Yes

If applicable, is the statistical analysis and its interpretation appropriate?

Yes

Are all the source data underlying the results available to ensure full reproducibility?

Yes

Are the conclusions drawn adequately supported by the results?

Yes

Are sufficient details of methods and analysis provided to allow replication by others?

Yes

Reviewer Expertise:

Energy systems analysis; demand response with residential heat pumps; reduced-order building modeling; optimization

I confirm that I have read this submission and believe that I have an appropriate level of expertise to confirm that it is of an acceptable scientific standard.

## References

[1] : The Prediction of Thermal Loads in Building by Means of the EN ISO 13790 Dynamic Model: A Comparison with TRNSYS. *Energy Procedia* .2016;101: 10.1016/j.egypro.2016.11.025 192-199 10.1016/j.egypro.2016.11.025
